# Local Knowledge for Addressing Food Insecurity: The Use of a Goat Meat Drying Technique in a Rural Famine Context in Southern Africa

**DOI:** 10.3390/ani9100808

**Published:** 2019-10-15

**Authors:** Martín del Valle M, José Tomás Ibarra, Pablo Aguirre Hörmann, Roberto Hernández, José Luis Riveros F.

**Affiliations:** 1 Department of Animal Sciences, Faculty of Agronomy and Forest Engineering, Pontificia Universidad Católica de Chile, Av. Vicuña Mackenna 4860, 78220436 Macul, Santiago, Chile; mdelval@uc.cl; 2 *ECOS (Ecology-Complexity-Society)* Laboratory, Center for Local Development (CEDEL) & Center for Intercultural and Indigenous Research (CIIR), Villarrica Campus, Pontificia Universidad Católica de Chile, O’Higgins 501, 4930000 Villarrica, Araucanía Region, Chile; tucuquere.jti@gmail.com; 3 Millennium Nucleus Center for the Socioeconomic Impact of Environmental Policies (CESIEP) & Center of Applied Ecology and Sustainability (CAPES), Pontificia Universidad Católica de Chile, Av. Vicuña Mackenna 4660, 78220436 Macul, Santiago, Chile; 4 Superintendence of Environment, Santiago de Chile. Teatinos 280, 8th floor, 8340434 Santiago, Chile; pjaguirreh@gmail.com; 5 Department of Environmental Sciences and Renewable Natural Resources, Faculty of Agricultural Sciences, Universidad de Chile. Av. Santa Rosa 11315, 8820808 La Pintana, Santiago, Chile; callevarela@gmail.com

**Keywords:** *Chinkui*, dried goat meat, local knowledge, food security, Mozambique

## Abstract

**Simple Summary:**

The *Bairro Boroma* (Boroma neighborhood) in Mozambique’s northwest Tete Province is characterized by its inhabitants’ low protein intake. This is despite being located in the region of the country with the largest number of livestock and having animal husbandry as one of its most important economic activities. Lack of access to electricity is a challenge for the conservation and regular consumption of meat in *Bairro Boroma*. We explored the role of local knowledge about a salty smoked preparation called *chinkui*, which was often used in ancient times in *Bairro Boroma*. Through a questionnaire about familiarity with *chinkui* and passive observation of its traditional preparation, we found that: (1) although most *Bairro Boroma* goat herders knew what *chinkui* was, its consumption was not frequent among villagers; and (2) the type of animal used to prepare *chinkui* does not produce an amount of meat that ensures its use as a sustainable source of meat. We concluded that, by exploring knowledge transmission methods and choosing animals with different traits, *chinkui’s* traditional preparation could be considered as an alternative for increasing high-quality protein intake in the context of the area’s serious food insecurity issues.

**Abstract:**

Only 30% of households in *Bairro Boroma* (*Boroma* neighborhood) have a regular protein intake, mainly due to the lack of a proper cold chain. We analyzed the level of knowledge about a local dried meat called *chinkui*, examining the relationship between this knowledge and its value for strengthening local food security. Through surveys of *Bairro Boroma* goat herders (*n* = 23) about “*chinkui* awareness” and passive observation of *chinkui* preparation (*n* = 5) from local biotype goats, we found that *chinkui* was known to most goat herders (91.3%), but was used only irregularly, mainly because knowledge transmission has decreased over time. From passive observation, we found that the amount of dried meat obtained from an animal rarely exceeded a yield of 10% and its performance and safety depended on weather conditions and the absence of other animals in the area of preparation. It is, therefore, recommended to strengthen initiatives to increase the amount of *chinkui*, based on local knowledge, so as to enhance its frequency of consumption and the possibility of using it as a sustainable alternative source of protein.

## 1. Introduction

At the 1992 Rio Earth Summit, the international scientific community and policymakers acknowledged the inextricable link between biological and cultural diversity and the importance of documenting local knowledge for the sustainable management of natural resources [1]. Local knowledge is the cumulative body of knowledge and practices associated with natural resources, including livestock, that have evolved through adaptive processes and been handed down across generations (adapted from Berkes, F. et al. [2]). Since 1992, international sustainability goals, such as the 2020 Aichi Targets, have frequently highlighted the importance of recovering local knowledge systems and power-sharing to strengthen sustainable local foodways, especially in rural famine or poor contexts [3,4].

Located in southern Africa, the Republic of Mozambique has a population of close to 30 million people [5] of whom 65% live in rural areas [6]. An estimated 45% of Mozambicans live below the poverty line, with 59% of them concentrated in rural areas [7]. Estimates for 2015–2017 suggest that 30% of Mozambicans were undernourished [8]. In the case of children, 43% suffered from chronic undernutrition [9] and only 37% of infants younger than six months were exclusively breastfed in 2010 [10]. Micronutrient deficiencies also have an impact since 69% of under-5s are anemic and 74% are vitamin A-deficient, posing a particular risk to their growth, immunity and development [11]. This is especially significant for a country where, in the first half of 2014, approximately 3.3 million cases of malaria were recorded [12]. In Mozambique, malaria is the leading cause of anemia in children [13], a condition that can be counter balanced by incorporating iron from meat into their diets.

Food waste, which accounts for one-third of the total food produced worldwide, has the greatest negative impact in the case of people facing food insecurity [14]. Food preservation techniques play a critical role in strengthening food security in these contexts, where the lack of access to electricity and a proper cold chain imply a permanent risk of food decomposition as a result of bacteria and fungi, threatening people’s health [15]. In drying processes, the color, shape and texture of meat may vary but most of its nutritional properties, including protein content, remain unchanged [16]. However, because it is not possible to halt the fatty acid rancidity process, the use of animal species characterized by lean meat is preferable. Goats are, therefore, one of the main livestock species used in protein preservation techniques for two main reasons: (1) their meat properties in terms of nutritional value (high levels of protein and minerals such as Ca and Fe) [17] and lean characteristics as well as (2) their availability and adaptability to arid zones [18].

In this paper, we assess the extent of use and local knowledge of a salty and smoked meat preparation known as *chinkui* in Mozambique’s northern Tete Province. We also discuss the potential of this local knowledge to enhance the access of the local rural population to an alternative/complementary source of protein, considering the important role meat sources can play in providing protein-rich food for undernourished people in developing countries [19]. We conducted this study using mixed, quantitative and qualitative approaches that allowed us to show the potential that local knowledge can have for strengthening food security in a rural famine context.

## 2. Materials and Methods

The study was conducted between 2016 and 2017 in the *Bairro Boroma* (Boroma neighborhood) of the town of Chitima (15°43′58.78″ S; 32°46′7.27″ E) in the Cahora Bassa District of Mozambique’s Tete Province. This province has the country’s largest number of caprine livestock (16.7%) [20] and has a dry tropical climate with annual rainfall that averaged 147.5 mm in 2014–2016 [21]. The *Bairro Boroma* community comprises approximately 190 families whose main economic activities are agriculture, animal husbandry and brick production. Most households are organized under community leaders, who define matters that include the kind of participation *Bairro Boroma’s* inhabitants have in external organizations, such as government public services or NGO projects [22].

### 2.1. Household Surveys: Local Knowledge of Chinkui

We designed and implemented a questionnaire of eight open and 14 closed questions related to “*chinkui* awareness” in order to explore the community’s level of knowledge about this meat drying technique and how it is valued. These questions included data on techniques, periodicity of consumption and the possible benefits of *chinkui* for family nutrition. The survey selection criteria were limited to goat herders in *Bairro Boroma*. The questionnaire was drawn up in Portuguese and the questions translated into *Nyungwe* or *Nhúngue*, the local *Bantu* language, with the help of a local translator. The goat herders signed a Free Prior Informed Consent before application of the questionnaire.

### 2.2. Passive Observation and Measurements of Chinkui Preparation from Local Biotype Goats

During our study period in *Bairro Boroma*, four local male nine-month-old and one seven-month-old goats were selected by the community to be slaughtered. We measured *ante* and *post mortem* data such as live weight, height and body condition and defined the carcass as the animal without hooves, tail, head, skin and internal organs, considering all pieces of meat from each animal, but without distinguishing by shoulder, leg and loin, among others when cutting. The slaughtering, cutting, piecing and drying processes were carried out by the community while we recorded aspects that could affect the quality of the final product, such as its exposure to pollutants. Cutting and pieced was made immediately post-slaughter. The cutting process was made by separating limbs from the body. Then, meat from limbs and the rest of the animal was removed separately. After that stage, meat was pieced and spent 12 h at 4 °C. Finally, the weight of *chinkui* was recorded to compare and estimate its yield.

## 3. Results and Discussion

### 3.1. Is Chinkui Pervasive in Bairro Boroma?

**Figure 1:**
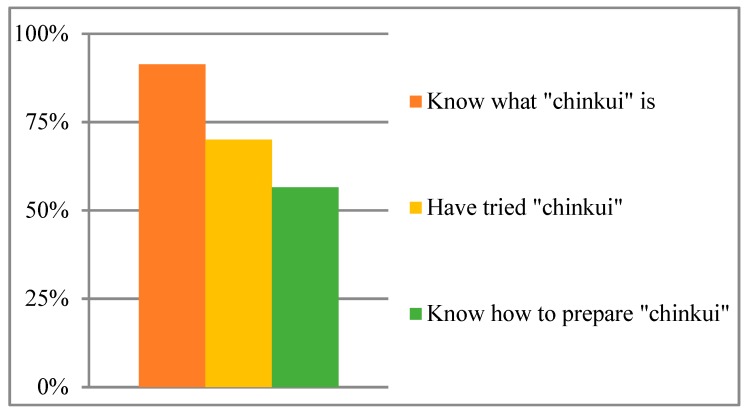
Knowledge of Bairro Boroma goat herders about chinkui.

*Bairro Boroma* had 25 goat herders of whom we interviewed 23. Out of the herders, 70% were men. All *Bairro Boroma* goat herders kept their animals in their household’s backyard and each member of the family had different duties associated with livestock management. Out of the 23 goat herders interviewed, 91.3% indicated that *chinkui* is a type of dried meat that can be prepared from different animal species, principally goats (34.7%) and bovines (26.7%). However, only 70% had consumed it at some point in their lives, while the remaining 30% had never tasted it. Asked if they knew how to prepare it, 56.5% indicated that they did (Figure 1). We found that consumption of *chinkui* over the years has been irregular: only 26% had eaten it during the previous five years; 13% had consumed it for the last time more than five years previously; and 61% did not remember. This irregularity could explain why it has a lower incidence in *Bairro Boroma* households’ diets.

*Chinkui* preparation starts with the slaughter of the animal and then progresses through meat piecing and preparation to final consumption. According to 84% of goat herders, it is prepared by cutting the meat into strips and boiling, salting and drying them using the sun’s heat. The remaining 16% indicated a preparation method that involved hanging the strips to dry in the wind and smoke from a fire. Those who opted for the “sun’s heat alternative” had homes with a solid roof while the smoking method was used by villagers who had a thatched roof or were working outside the village and had to sleep in the bush. Both techniques were based on the same principle, which is illustrated by Rahman et al. [23]: dehydration of meat from the center to the periphery to eliminate the moisture needed by bacteria and fungi in order to grow. As regards responsibility for *chinkui* preparation within households, this did not represent a unique duty because the person who prepared it was usually also responsible for cooking all kinds of food. According to 69.2% of goat herders, the same person had always supervised this work in their household, with no differences by gender.

In terms of the knowledge’s origin, 80% of those who knew how to prepare *chinkui* had received instruction from their parents or grandparents, but only 30% taught their children how to prepare it, focusing on male children (57%). According to 52.7% of the herders, the way of teaching how to make *chinkui* had not changed over time. Only 30% indicated that they knew about other methods of preparation, such as fire-drying or the use of a gas oven, while the remaining 70% had always prepared *chinkui* in the same way. Even if information about “how to prepare it” exists, this cooking technique requires constant experience of it in order to achieve cultural acceptance and appropriation, apart from the nutritional benefits it may have, and must be constantly discussed to ensure its sustainability over time. This is related to what Weichselgartner and Pigeon [24] describe as the “dynamic” nature of knowledge since this set of practices needs to be performed regularly in order to be culturally acceptable and handed down through the generations.

According to 78.2% of participants, consumption of *chinkui* among men and women is similar, with 86.9% of interviewees indicating that no-one is banned from consuming it while the remaining 13.1% either did not know or did not answer. According to 78.2%, *chinkui* has no ceremonial value, while the remaining 21.8% indicated that *chinkui* can be served at funerals as something to offer to relatives and friends. This differs, for example, from the Maasai culture where some special foods, including dried meat, are given to boys and girls post-circumcision [25].

In relation to local evaluation of the value of *chinkui* as a nutritional source, 82.6% of goat herders considered it a “very nutritious” food. According to them, reasons such as “it does not rot” (39%), it has a “good taste” (23%) and “well-prepared meat is healthy” (38%) add value and allow the animal to be considered a source of protein. In this way, goat herders pointed out, it makes the body “healthy and strong”. Thus, 91.3% considered the incorporation of *chinkui* into their family’s diet as positive for their well-being.

This suggests that a food’s perceived nutritional attributes are related to aspects that have to do with the ability to preserve it and its taste, in addition to the chemical properties it possesses. Finally, the local importance of *chinkui’s* characteristics may be valuable for future efforts to improve local livestock production and for nutritional interventions in the *Bairro Boroma* community.

### 3.2. Passive Observation of Chinkui Preparation from Local Biotype Goats

**Figure 2:**
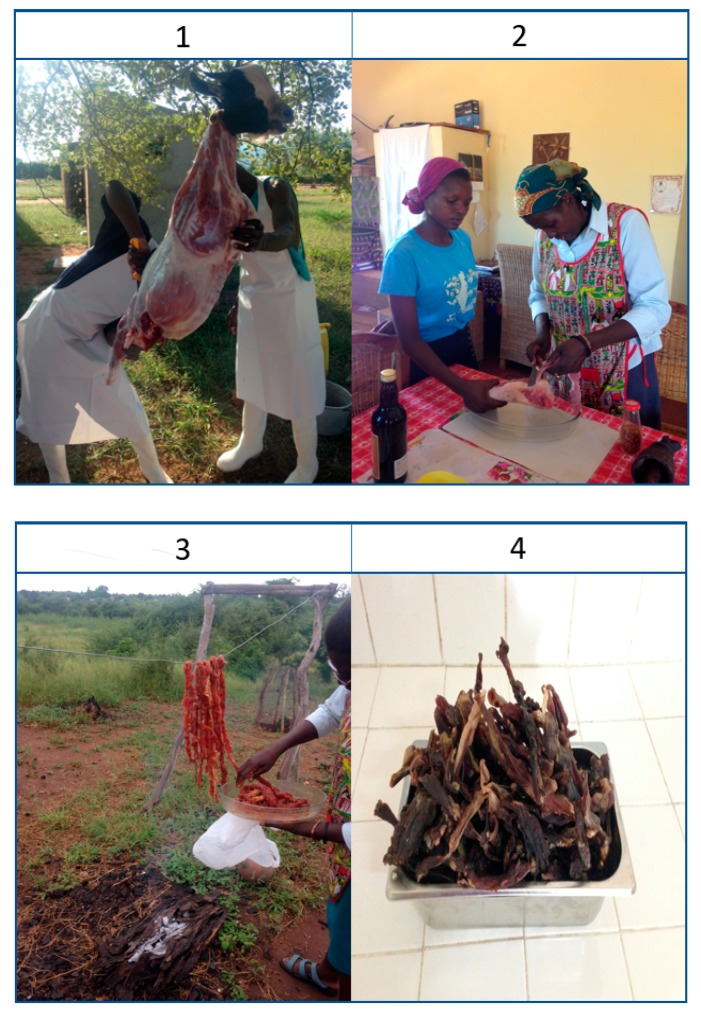
Stages of chinkui preparation: (1) Slaughtering; (2) Cutting and seasoning; (3) Strips hung out to dry; (4) chinkui dried meat.

We found that *chinkui* preparation starts by removing the meat from the carcass in the form of standard strips that are approximately 20 cm long, 1 cm high and 2 cm wide. Recipe guidelines then indicate a second step when it is seasoned with salt, red wine and garlic and left during the night. The third step involves hanging the seasoned meat strips over a small fire until the next morning to be dried by the air and smoke. After 30 h, the product is ready for consumption. This process requires constant attention, principally because of the presence of birds, bugs, mice and snakes that can eat and contaminate the meat, posing a health risk for its consumption. In addition, the process requires that it is not raining. Figure 2 shows the main stages of *chinkui* preparation.

With the information about the weight of *chinkui* produced, we could organize the data for each animal in order to compare results and discuss this technique from a productive point of view. Table 1 shows *ante* and *post mortem* characteristics and the *chinkui* weight for each animal during the passive observation.

The percentage of dried meat (*Chinkui* vs. Live Weight) exceeded 10% for only one of the five animals. This could mean that, if working exclusively with young local biotypes, the amount of meat obtained may be a limiting factor, considering the alternative value that goats have as a financial instrument for obtaining other goods in rural Africa [26]. The introduction of specialized breeds and hybridization with them, while also working with older animals, is an approach that could enhance productive parameters without the loss of the rusticity necessary for adaptation to territories with unfavorable agro-climatic characteristics [27]. This approach may be important for increasing regular access to good quality protein.

The 10% yield may seem poor when *chinkui* and LW are compared. However, when the amount of *chinkui* is compared with the amount of raw meat, the figure rises to 35.5%, very similar to the 32% yield of the South African *biltong* [28], but still well below the Brazilian *carne-de-sol*, whose average yield can reach 50% [29]. However, it is important to note that this technique, based in a drying process and the use of salt, permits the preservation of the meat as a stable and safe source of protein, iron and other micronutrients, mainly due to the decrease of water activity [30]. In addition, its cultural roots and the fact that drying and fermentation have been reported as the oldest methods for food preservation [31], could facilitate its incorporation into a future dietary intervention, especially in a context of low food security indicators such as those seen in *Bairro Boroma*. Nevertheless, future research is needed to identify an appropriate way to ensure its sustainable incorporation at the household level.

## 4. Conclusions

*Chinkui* is widely known by *Bairro Boroma* goat herders as a “dried meat” preparation, although knowledge about how to prepare it and the transmission of this knowledge across generations seems to have decreased in recent years. Nevertheless, besides its potential value as a rich source of protein in an undernourished context, its recovery might be relevant for two main reasons: (1) it is a product highly valued by the community because of its “taste” and “durability” and because it is meat; and (2) it has cultural roots that could facilitate its incorporation into *Bairro Boroma* households’ diets.

The yield obtained from the trial suggests a need to explore means of increasing the amount of meat obtained in line with the characteristics of the territory (cultural and climatic). Together with the innocuous characteristics contributed to *chinkui* by the drying process, it could be considered an important nutritional source in a context of food insecurity such as *Barrio Boroma*.

The disconnection between livestock and protein intake in a region with the largest number of goats in Mozambique could be addressed by exploring alternatives for food preservation based on local knowledge, such as *chinkui*. We propose to focus future applied research on exploring innovative forms of community engagement and empowerment to ensure the sustainability of this technique and to evaluate its use as a direct source of protein in local households.

## Figures and Tables

**Table 1:** Ante mortem, post mortem and chinkui production characteristics recorded through participant observation.

Goat Number	Declared Age (months)	Live Weight [LW] (kg)	Height (cm)	Body Condition (1–5)	Hot Carcass Weight (kg)	Pieced Meat (kg)	*Chinkui* (kg)	*Chinkui* vs. LW (%)
**1**	9	25.14	54	3	9.2	4.8	2.1	8.2
**2**	9	16.2	50	3	7.2	4.6	1.7	10.1
**3**	9	19.7	51.5	4	6.9	4.8	1.3	6.7
**4**	9	18.3	49.5	3	7	4.3	1.4	7.4
**5**	7	21.8	58	3	8.6	4.3	1.6	7.2
**Average**	8.6 ± 0.9	20.2 ± 3.4	52.5 ± 3.5	3.2 ± 0.5	7.8 ± 1	4.6 ± 0.3	1.6 ± 0.3	
